# Editorial: How the application of antimicrobial hurdles in meat processing facilities shapes microbial ecology

**DOI:** 10.3389/fmicb.2024.1501925

**Published:** 2024-11-08

**Authors:** Xianqin Yang, Michael Gänzle, Rong Wang

**Affiliations:** ^1^Agriculture and Agri-Food Canada Lacombe Research and Development Centre, Lacombe, AB, Canada; ^2^Department of Agricultural, Food and Nutritional Science, University of Alberta, Edmonton, AB, Canada; ^3^U.S Meat Animal Research Center, Agricultural Research Service (USDA), Clay Center, NE, United States

**Keywords:** selective pressure, microbial ecology, persistence, biofilm, sanitation

Meat production, i.e. converting livestock into meat, is a complex process, not only in that it has many steps in the process, but also in that facility has a flow of animals, water, air, and workers, all of which serve as carriers of bacteria. Livestock, for instance, may harbor up to 10^11^ CFU/g feces (Dowd et al., 2008) and up to 10^10^ CFU/cm^2^ bacteria on their hides (Yang, 2017). Some of those bacteria are human pathogens, without causing overt disease in their animal host such as Shiga toxin-producing *Escherichia coli* (STEC), *Salmonella*, or *Campylobacter*. A high proportion of healthy animals presented for slaughter may carry these pathogens on their hides/skin or in their intestines or lymph nodes (Zhang et al., 2024; Arthur et al., 2007). Consequently, antimicrobial interventions such as spraying hide- on carcasses with sodium hydroxide, pasteurizing carcasses with hot water and spraying carcasses with various organic acids have been widely implemented in the carcasses dressing process to control pathogens (Gill, 2009; Loretz et al., 2011). Many studies have evaluated the efficacy of these treatments for pathogen reduction and hygiene indicators. However, in addition to these antimicrobial interventions, other factors also play important roles in shaping the microbial ecology in meat processing environment by exerting selective pressure, including but not limited to operation temperatures, relative humidity, routine cleaning and sanitation of the facility, and difficult to access places by cleaning effort in equipment. This Research Topic collected five articles that add to our understanding of how antimicrobial hurdles shape the microbial ecology in meat processing facilities and meat products as well as their relationship with antimicrobial resistance.

Shiga toxin producing genes in *E. coli* are encoded on prophages integrated into the bacterial chromosome (Ohnishi et al., 2001). The production of Shiga toxins can be induced as a response to stressful conditions. Castro et al. investigated the potential of 48 *E. coli* isolates with intact or fragmented *stx*_1*a*_ for Shiga toxin production under conditions relevant to foods. Production of Shiga toxins was observed only for *E. coli* isolates that carried the complete *stx* gene (*n* = 11). They reported down-regulation of the toxin production by acidic conditions and lethal temperatures, and favorable toxin production by neutral pH and milk, and incubation at 40°C for some strains, the latter of which may be linked to a greater diversity of the promotor regions of Stx-prophages, and of genes related to cell adhesion and stress tolerance.

Dittoe et al. examined the effect of treating raw poultry products with organic and inorganic acids on the meat microbiota. They reported that chicken wings treated by a 15-s dip in organic acid (peroxyacetic acid; PAA), inorganic acid (sodium bisulfate; SBS), or their combination (PAA + SBS) had similar total bacterial count by 21 days of chiller storage. However, wings treated with SBS and SBS+PAA had a 7-day shelf life advantage over wings that were treated with tap water, and the treated wings had lower relative abundance of typical spoilage populations while having a greater relative abundance of *Bacillus* spp. These findings suggest that antimicrobial interventions differentially affect the meat microbiota and that desirable shifts in the composition of microbial communities on meat can be exploited for shelf life advantage.

**Figure 1:**
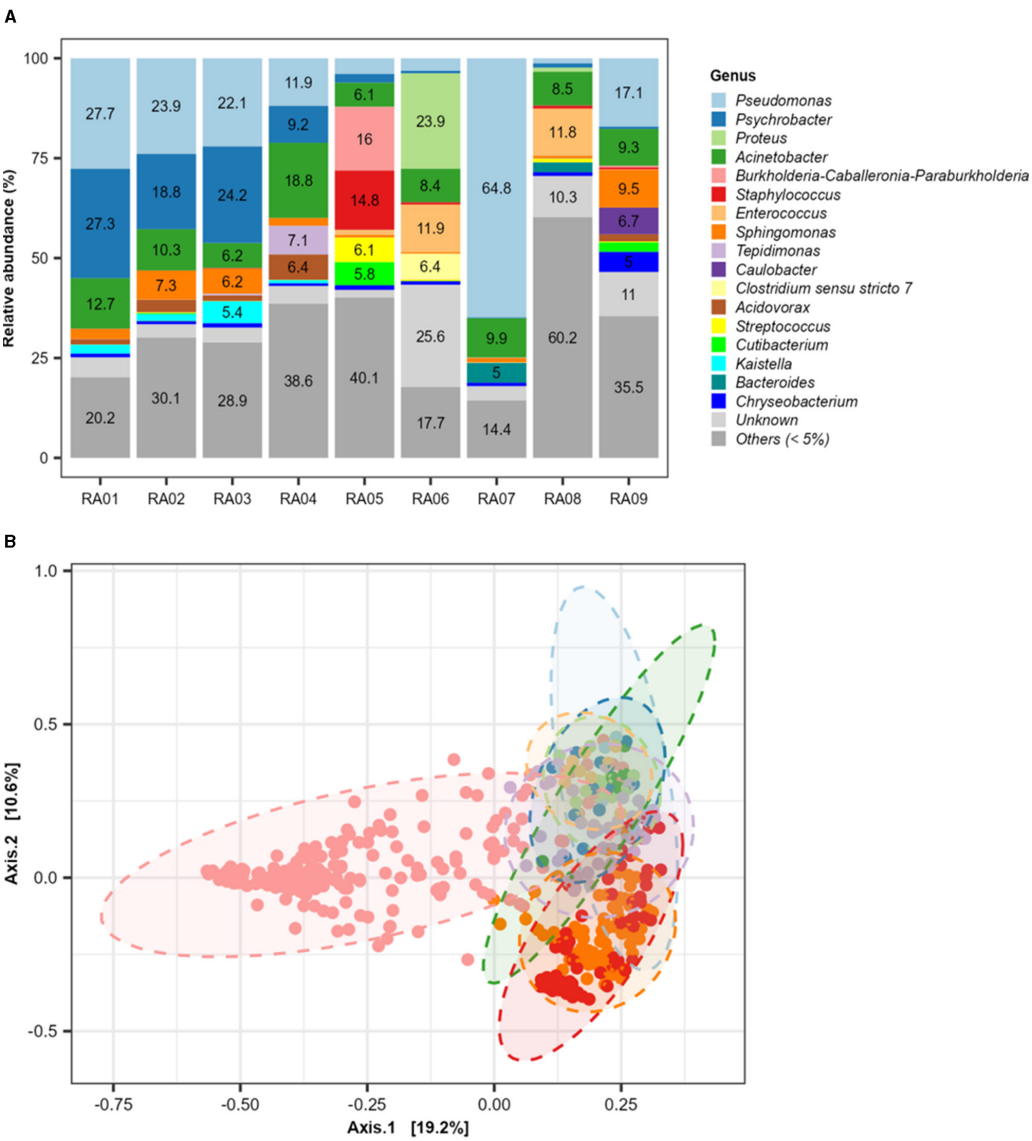
Diversity of persistent bacterial genera in processing plants. **(A)** The relative abundance of bacterial genera in different functional rooms (RA, room after cleaning and sanitation); **(B)** Principal coordinate analysis (PCoA) plot distinguished with room numbers. This is adapted from Yang et al.

Yang et al. reviewed the potential factors influencing the microbial ecology in commercial meat processing facilities and conducted a meta-analysis on the microbiota data published in the last 10 years. Some *E. coli* strains achieved persistence on post-sanitation equipment surface, likely through biofilm formation and difficult to clean harborage site, rather than resistance to biocides of their planktonic cultures. The authors also reported the persistence of diverse bacteria in meat plants in genus level, *Pseudomonas, Acinetobacter, Psychrobacter, Sphingomonas, Enterococcus, Proteus, Staphylococcus, BurkholderiaCaballeronia-Paraburkholderia, Acidovorax*, and *Brevundimonas* (Figure 1). These non-pathogenic bacterial strains may also enhance the biofilm formation of foodborne pathogens who otherwise do not form biofilms on their own, suggesting targeted cleaning and sanitizing efforts against residential microbiota may be rewarding in both safety and storage stability of meat products.

Koti et al. investigated the impact of temperature and companion bacteria including lactic acid bacteria (*Carnobacterium piscola* and *Lactobacillus delbrueckii* subsp. *bulgaricus*) and spoilage bacteria (*Comamonas koreensis, Raoultella terrigena*, and *Pseudomonas aeruginosa*) on the susceptibility to biocides of STEC in planktonic cultures as well as in biofilms. In general, planktonic cultures and single species biofilms exhibited greater susceptibility to all biocides tested (quaternary ammonium compounds, sodium hypochlorite, sodium hydroxide, hydrogen peroxide, BioDestroy). Interestingly, all STEC strains tested had higher counts in multispecies biofilms with *Raoultella* sp. and *Comamonas* sp. than with *Carnobacterium* sp. and *Lactobacillus* sp. or *Pseudomonas* sp. and *Comamonas* sp. The extent of reduction of STEC by biocides is a function of temperature, the companion bacterial strain as well as the STEC strain.

Pathogen contamination incidence in the meat industry is often addressed by intense sanitization (IS) of the entire processing plant. Wang et al. examined the immediate and long-term impact of such sanitation on the environmental microbial community and pathogen colonization. They reported that even though the colonization of *Salmonella* in drains did not differ between pre- and post-IS biofilms, post-IS samples formed stronger biofilms and in certain cases resulted in better *Salmonella* survival in response to sanitizers. The alteration in microbial community structure may be related to stronger biofilm formation of post-IS drain samples through survival, recruitment and overgrowth of species with high colonizing capability.

In conclusion, the five articles in this Research Topic better our understanding of factors shaping the microbial ecology of meat processing facilities and mechanisms of persistence, especially for pathogenic organisms. Bacterial activities and persistence are often strain dependent (Xu et al., 2024). Future *in-situ* studies with strain level resolution of presence and interactions would advance our understanding of this area even further.
